# Post-Translational Modification Crosstalk in Gastric Cancer: Molecular Switches, Signaling Networks, and Therapeutic Opportunities, with Comparative Insights from Colorectal Cancer

**DOI:** 10.3390/cancers18193107

**Published:** 2026-09-24

**Authors:** Jun Wang, Xianying Chen, Yu Wang, Yuehua Han

**Affiliations:** 1 Department of Gastroenterology Surgery, The Second Affiliated Hospital, Zhejiang University School of Medicine, Hangzhou 310000, China; 2 Department of Pathology and Pathophysiology, School of Basic Medical Sciences, Zhejiang University, Hangzhou 310000, China; 3 Department of Gastroenterology, The Second Affiliated Hospital, Zhejiang University School of Medicine, Hangzhou 310000, China

**Keywords:** post-translational modification, PTM crosstalk, gastric cancer, ubiquitination, SUMOylation, lactylation

## Abstract

Gastric cancer remains a leading cause of cancer-related death, and its malignant behavior is driven in large part by dysregulated post-translational modifications (PTMs). Yet the prevailing tendency to study individual modifications in isolation has obscured a defining feature of PTM biology: modifications frequently interact, competing for the same residue or cooperating across residues to form regulatory switches that dictate protein fate. This review synthesizes the PTM crosstalk framework in gastric cancer around three principal axes—phosphorylation–ubiquitination relays, SUMOylation–ubiquitination interplay, and metabolic coupling through lactylation—and traces how these axes translate into the hallmarks of proliferation, metastasis, therapeutic resistance, and immune evasion. The framework is further extended to colorectal cancer to separate shared regulatory principles from cancer-specific switches. Central to this account is the proposition that oncogenic output is governed not by any single modification but by the balance among competing and cooperating marks, and that the switch enzymes maintaining this balance constitute the most informative therapeutic targets. Translating this view into therapy will require selective degraders and inhibitors, crosstalk-aware biomarkers, and systematic multi-omics maps of the modification network.

## Simple Summary

Gastric cancer is a deadly disease, and its growth and spread are driven by chemical “tags” that are added to or removed from proteins inside cancer cells. Scientists usually study each tag on its own, but the tags do not act alone: several tags on the same protein can compete or cooperate, and together they act as molecular switches that decide whether a cancer-driving protein is switched on or destroyed. This review organizes these tag-to-tag interactions into three main types and shows how they control the growth, spread, drug resistance, and immune escape of gastric cancer, with comparison to colorectal cancer. We propose that targeting the enzymes that control these switches—rather than any single tag—offers new opportunities for treatment and could guide the development of future drugs and tests.

## 1. Introduction

Gastric cancer is a leading cause of cancer-related death worldwide [1], and even advanced or metastatic cases have limited treatment options and a poor prognosis. Although targeted therapy and immune checkpoint blockade have reshaped the treatment landscape for some patients, prolonged responses are still limited to a small proportion of the population, so the molecular circuits responsible for the sustained development, spread, and treatment resistance of gastric cancer need to be identified. Protein post-translational modifications (PTMs), such as phosphorylation, ubiquitination, acetylation, methylation, SUMOylation, and glycosylation, regulate protein stability, activity, location, and interaction, and their dysregulation is associated with cancer [2]. Among these, ubiquitination has long been recognized as a central regulator of protein turnover since the characterization of the ubiquitin system [3]. Aberrant PTMs in gastric cancer promote cell proliferation, migration, metastasis, and chemotherapy resistance, making the PTM machinery a frequent oncogenic driver. For example, the E3 ligase MDM2 degrades somatostatin and thereby inhibits CD8+ T-cell function [4]; deubiquitinases work in the opposite direction, with USP48 stabilizing HEG1 to promote AKT1 [5] and JOSD1 increasing SULF1 levels to activate Wnt7B-FZD1 signaling [6]; and the deSUMOylase SENP1 modifies ENO1 to alter glycolysis and confer cisplatin resistance [7]. However, most existing reviews have catalogued individual PTMs in isolation—phosphorylation in one section, ubiquitination in another, SUMOylation in a third [8]. Even recent comprehensive reviews of PTMs in gastric cancer, which do acknowledge crosstalk, still treat it as a secondary topic rather than an organizing principle [9]. Although this modification-by-modification scheme is convenient for listing enzymes and substrates, it obscures that modifications do not occur in isolation: multiple modifications often occur simultaneously on a single protein and can compete or cooperate to regulate its function [10]. Only recently have researchers begun to study this interaction of modifications—PTM crosstalk—systematically in gastric cancer. SUMOylation and ubiquitination converge on shared lysine residues to fine-tune the stability of oncogenic regulators, as exemplified by TRIM28-orchestrated SUMO–ubiquitin crosstalk in bladder cancer [11], and lactylation can prevent the degradation of substrates such as S100A11 to promote gastric cancer metastasis [12], while lactate-induced histone modifications promote immune evasion [13]. These observations indicate that the functional output of the PTM network in gastric cancer is governed not by a single modification but by competition and cooperation among multiple modifications. This review examines PTM crosstalk in gastric cancer and, by considering colorectal cancer, also investigates the shared regulatory mechanisms and cancer-specific switches. We first introduce the general categories of crosstalk, then review the crosstalk axes that govern oncogenic signaling and cancer hallmarks in gastric cancer, and finally discuss therapeutic strategies targeting the “switch” enzymes at the core of these networks. Rather than listing modifications one by one, this review adopts a network perspective to clarify how PTM crosstalk shapes the biology of gastric cancer and to identify conceptual and translational opportunities that single-modification studies have overlooked. The literature was identified through searches of PubMed and Google Scholar for the terms “gastric cancer,” “colorectal cancer,” “post-translational modification,” “crosstalk,” “ubiquitination,” “SUMOylation,” “phosphorylation,” and “lactylation,” without restriction on publication date, supplemented by citation tracking of key primary studies. Because the review is narrative rather than systematic, the selected studies are illustrative of each crosstalk mode rather than exhaustive, and priority was given to mechanistic, original research articles. Beyond this organizational shift, the review contributes three specific advances over prior syntheses: first, it distinguishes authentic inter-modification crosstalk from single-modification regulation, a distinction that is frequently blurred in the literature; second, it integrates lactylation explicitly into the crosstalk framework as a metabolic–ubiquitin interface rather than treating it as a standalone epigenetic mark; and third, it provides a cross-cancer comparison that separates conserved gastrointestinal mechanisms from gastric-cancer-specific switches.

## 2. The Concept and Modes of PTM Crosstalk

For a long time, protein post-translational modifications have been considered to be independent, residue-specific regulators of protein activity, stability, location and interaction. However, the single-modification model is gradually being regarded as insufficient; multiple modifications often occur at the same protein and are functionally related, and ultimately, it is the combination of these marks—not any single mark in isolation—that determines the protein’s fate. PTM crosstalk refers to three kinds of recurring phenomena: competition among modifications at the same residue, cooperation between distant residues, and metabolic or epigenetic coupling that connects the cell’s condition to modification status (Figure 1). Throughout this review, we reserve the term “crosstalk” for events in which two or more distinct modifications functionally interact on a protein—whether by competing for the same residue, by one modification priming recognition by an enzyme of another, or by a metabolite-derived mark altering the readout of the ubiquitin system—and we explicitly flag examples that instead reflect regulation by a single PTM enzyme, so that genuine inter-modification interplay is not conflated with single-modification signaling. The following modes are introduced to provide a unified vocabulary for discussing the oncogenic signalling network of gastric cancer.

### 2.1. Same-Residue Competition

Same-residue competition is a straightforward way: two or more modifications compete for a single amino acid, and thus, if one is present, the other is excluded, forming a binary switch. Lysine residues are the main sites for such competition because they are substrates of ubiquitination, SUMOylation, acetylation, methylation and lactylation; therefore, occupancy by one modification prevents the others and directs the protein towards degradation, stabilisation, relocalization or altered activity. The examples that follow are drawn from general cell-biology precedents that established this mode mechanistically, and they serve here as the conceptual foundation for the gastric-cancer-specific switches detailed in Section 3. The SUMOylation–ubiquitination axis is a typical example: TRIM28 shifts its substrates between SUMOylation and ubiquitination to regulate their stability [11]. Acetylation can likewise oppose ubiquitination: acetylation of ATP-citrate lyase at K540, K546, and K554 blocks its ubiquitination and prevents degradation [14]. SUMOylation and ubiquitination also converge on shared substrates: MYC is SUMOylated at K51 and K52 by the SUMO E3 ligase PIAS1 [15] and is governed by both modifications [22], and even the lysine-less tumor suppressor p14ARF is stabilized by N-terminal SUMOylation that opposes ubiquitin-driven degradation [23]. Beyond lysine-directed competition, glycosylation on other residues can also antagonize ubiquitination: the N-linked glycan of ABCG2 protects the transporter from ubiquitin-mediated degradation [24], and removal of N-linked glycosylation is a prerequisite for MDM2-mediated ubiquitination of PD-1 [25]. Lactylation can likewise protect substrates from degradation: lactylation of S100A11 shields the protein from degradation and thereby promotes gastric cancer metastasis [12]. In all these cases, competitive crosstalk acts as a molecular “switch” whose output is set by the relative amounts and activities of the competing writers and erasers. Because the two modifications are mutually exclusive at a given site, the informative quantity is their ratio rather than the absolute level of either mark; measuring only one modification will miss the functional state of the substrate, and it is this ratio that is most likely to correlate with oncogenic behavior.

### 2.2. Cooperative Crosstalk Across Residues

Cooperative crosstalk across different residues forms a second type in which one modification creates or removes a recognition motif that directs a second modification, thus producing a sequential cascade. The best-known representative is phosphorylation-dependent ubiquitylation; that is, a kinase phosphorylates a substrate to create a phosphodegron that is recognized by an E3 ubiquitin ligase and thus tagged for proteasomal degradation. The canonical β-catenin pathway is a typical example of this phosphorylation–ubiquitination relay; GSK3β-mediated phosphorylation primes β-catenin for recognition by the SCFβ-TrCP ligase and subsequent degradation [16,17] (Figure 2). Cooperation is not limited to degradation; acetylation and methylation can also generate docking sites for ubiquitin ligases and deubiquitinases, thereby propagating signaling. Acetylation is a type of bidirectional regulation: acetylation of PSAT1 promotes its recognition by the E3 ligase UBE4B and subsequent proteasomal degradation [18], while acetylation of TRIM25 enhances its interaction with the deubiquitinase USP7 and thus stabilizes the protein [19]. Methylation-related enzymes are also connected to the ubiquitin system: EZH2 recruits USP7 to stabilise LSD1, DNMT1 and β-catenin [26], and LSD1-mediated demethylation of HIF-1α protects the protein from ubiquitination-dependent degradation [27]. Crosstalk can also connect the two main degradation pathways; for instance, arginylation (addition of an arginine residue) and sequential ubiquitination of AKT target it for autophagic degradation [28]. The functional result of cooperative crosstalk is a “cascade” switch, in which the modification history of the protein itself—rather than a single fixed mark—encodes its signalling status [10]. Because each step depends on the preceding one, the sequence acts as a ratchet that commits the substrate to a given fate once the initial modification is made, distinguishing a genuine cascade from mere co-occurrence of marks and converting a transient upstream cue into an irreversible downstream decision such as degradation.

### 2.3. Metabolic and Epigenetic Coupling

Metabolic and epigenetic coupling are a third type; that is, alterations in metabolites within the cell directly alter the function of proteins and chromatin. Among the metabolite-derived modifications, lactylation is singled out here not because it is the only such mark, but because it is the one for which the most direct links to the glycolytic phenotype of gastric cancer, to ubiquitin-system function, and to immune evasion have been established; succinylation and crotonylation follow the same principle but have fewer gastric-cancer-specific demonstrations to date. Lactylation is a typical recent example: lactate, which originates from the glycolytic pathway, is covalently attached to lysine residues on histones and non-histone proteins to regulate gene expression and immune regulation [20]; this addition is carried out by lactyltransferases, such as the ACSS2–KAT2A axis [30]. Lactate-driven histone lactylation remodels immune evasion in gastric cancer via KDM5B-mediated histone demethylation [13], and lactylation can also orchestrate ubiquitin-independent degradation of the DNA sensor cGAS to promote tumour growth [31]; thus, a metabolite serves as a substrate for the PTM network. Succinylation follows the same principle in gastric cancer: CPT1A-mediated succinylation of S100A10 at K47 suppresses its ubiquitination and degradation, thereby promoting gastric cancer invasion [21]. Other metabolite-derived acylations, such as crotonylation, are similarly regulated by metabolite availability. This coupling mode is conceptually different from competition and cooperation because its input is environmental or metabolic, not enzymatic antagonism; it can explain how the PTM network senses and responds to the metabolic status of a cancer cell. This sensing capacity is what makes metabolic coupling clinically relevant: it means that the modification landscape is not a fixed property of the cancer cell but is continuously shaped by the tumor’s metabolic state, and therefore by its nutrient supply, oxygenation, and glycolytic activity—factors that are themselves dynamic during tumor growth and treatment. Competition, cooperation and metabolic coupling thus form a unified framework for interpreting PTM crosstalk in gastric cancer. These three modes are not mutually exclusive—a single protein can participate in a competitive toggle and a cooperative cascade at once, and a metabolic mark such as lactylation can feed into both—and each maps onto a distinct therapeutic logic: competitive toggles can be modulated by inhibiting the dominant writer or activating its eraser, cooperative cascades by interrupting the recognition step, and metabolic coupling by targeting a metabolite or its ligase. The field nonetheless remains constrained by a bias toward single-protein, single-modification studies, and systematic, proteome-scale maps of crosstalk are still lacking, particularly in gastric cancer. How these modes are combined in oncogenic signalling networks, how they differ in gastric and colorectal cancers, and how they can be used therapeutically are the problems addressed in the following sections.

## 3. Crosstalk Axes Governing Oncogenic Signaling in Gastric Cancer

Oncogenic signalling in gastric cancer is governed by interconnected modification axes that regulate key substrates. The first two axes couple two modifications on the same protein—phosphorylation with ubiquitination, and SUMOylation with ubiquitination—whereas the third, lactylation, is a metabolite-derived mark that couples cellular metabolism to protein function and chromatin. Each architecture regulates the stability, activity or localisation of key oncogenic regulators, and together they form a circuit that drives proliferation, survival, metabolism and immunity. A common theme runs through these axes: the fate of an oncoprotein is set not by a single mark but by the interplay among modifications and by the metabolic state that feeds them. Two consequences follow: the functional state of an oncoprotein cannot be read from a single mark, and the most informative therapeutic targets are the enzymes that regulate these balances.

### 3.1. Phosphorylation–Ubiquitination Relays

Phosphorylation and ubiquitination form a cooperative relay in which a kinase phosphorylates a substrate to create a phosphodegron that an E3 ligase then recognizes for ubiquitination and proteasomal degradation (Figure 2). In gastric cancer this relay is exemplified by CK1δ, which phosphorylates ATF4 to trigger its β-TrCP-dependent ubiquitination and degradation, thereby promoting chemoresistance [29]. The same phosphodegron logic underlies the canonical Wnt pathway, in which β-catenin is phosphorylated and then ubiquitinated for degradation [16,17]. Through this phosphorylation-dependent ubiquitination, kinase activity is directly converted into protein turnover, coupling upstream oncogenic signals to the proteolytic control of key regulators. This relay is efficient because a single kinase can control the stability of many substrates, and its specificity derives less from the kinase than from the E3 ligase that reads the phosphodegron; the same phosphorylated motif can therefore be interpreted differently in different contexts, converting a graded kinase signal into an all-or-none decision on protein abundance.

### 3.2. SUMOylation–Ubiquitination Interplay

SUMOylation and ubiquitination form a second crosstalk axis, because both modifications act on lysine residues and can oppose or license each other [32] (Figure 3). In gastric cancer this interplay runs in both directions. SUMOylation can oppose ubiquitination: TRIM28 switches PD-L1 from ubiquitination to SUMOylation—promoting SUMOylation while inhibiting ubiquitination—thereby stabilizing the immune checkpoint and driving immune escape [33]. SUMOylation can also license ubiquitination: SUMOylation of SREBP2 at K464 promotes its recognition by the E3 ligase FBXW7 for ubiquitin-dependent degradation, and SENP1-mediated deSUMOylation at K464 disrupts this interaction to drive de novo cholesterol synthesis [34]. The SUMO–ubiquitin axis therefore acts as a bidirectional switch, in which the presence or removal of SUMO determines whether a substrate is routed toward stabilization or degradation. Because SUMOylation and ubiquitination are both reversible and catalyzed by opposing enzyme families, the net fate of a substrate reflects a dynamic equilibrium that can be shifted by the abundance or activity of SUMO ligases, deSUMOylases, E3 ligases, or deubiquitinases—any one of which is a potential point of therapeutic intervention.

### 3.3. Lactylation: Metabolic-Epigenetic Coupling

Unlike the two preceding axes, which couple two modifications on the same protein, lactylation is a single metabolite-derived mark that couples the glycolytic state of the cell to protein function and chromatin. It nonetheless intersects with the ubiquitin system in two ways. First, lactylation can shield substrates from degradation: lactylation of PD-L1 at K280 inhibits binding of the E3 ligase HUWE1 and thereby prevents ubiquitination and proteasomal turnover, stabilizing the checkpoint and promoting immune evasion [36]; likewise, lactylation of S100A11 at K27 and K55 protects the protein from degradation to drive metastasis [12]. Second, lactylation can bypass ubiquitination altogether: lactylation of cGAS at K21 promotes its ubiquitin-independent proteasomal degradation, in which the proteasome subunit PSMA4 directly captures lactylated cGAS, suppressing the interferon response and promoting tumor growth [31]. Lactylation also remodels chromatin—H3K18 lactylation drives POM121 transcription [37], and CSDE1-mediated histone lactylation of the HOXD9 promoter supports progression [38]—and the mark is dynamically reversed, as HDAC-mediated delactylation (removal of the lactate-derived mark) of LDHB at K58 promotes metastasis through ferroptosis resistance [39]. Whereas the writer side of lactylation is increasingly well defined, the eraser side—the specific delactylases that remove this mark—remains less completely characterized: histone deacetylases have been implicated in delactylation, but their substrate specificity, isoform identity, and the extent to which delactylation is a regulated, signal-responsive process rather than a by-product of broad deacetylase activity are still unresolved. This asymmetry matters therapeutically, because a complete regulatory switch requires both arms, and the identity of the relevant delactylase may represent an independent target distinct from the lactyltransferases. Through this single metabolite-derived mark, lactylation therefore links the metabolic state of gastric cancer cells to protein stability, chromatin regulation, and immune evasion. Thus, although lactylation is itself a single modification rather than a two-modification coupling, its functional interactions with the ubiquitin system—shielding substrates from ubiquitination, bypassing ubiquitination, and dynamic delactylation—constitute a broader metabolic–ubiquitin crosstalk. Conceptually, lactylation is distinct from the two preceding axes because its upstream input is the metabolic state of the cell rather than the activity of an opposing enzyme: the availability of lactate, and hence the extent of lactylation, is set by glycolytic flux, so the modification acts as a sensor that couples nutrient and oxygen availability to protein and chromatin function. This property makes lactylation an especially attractive target in gastric cancer, whose glycolytic phenotype is well established, and it explains why the metabolic and the ubiquitin systems should be considered together rather than as separate regulatory layers. A common theme unites these three architectures: gastric cancer uses modification interplay and metabolic coupling to control the fate of proteins, pathway activities and chromatin structure, and the most tractable vulnerabilities are the switch enzymes—the competing writers, erasers and ligases—rather than any single modification. The current knowledge is nonetheless fragmented: most axes have been characterised for individual proteins in isolation, and systematic maps showing how these switches are integrated, rewired during metastasis and therapy resistance, and differ between gastric and colorectal cancers are yet to be established. Finally, the three axes are not independent pathways but shared nodes in a single regulatory web, because the same substrates—often the same lysine residues—can be acted upon by the phosphorylation, SUMOylation, ubiquitination, and lactylation machineries; this overlap makes crosstalk a network property rather than a set of pairwise interactions, which a modification-level view systematically underestimates.

The crosstalk network is also subject to hijacking by environmental carcinogens, most notably Helicobacter pylori, the principal bacterial driver of gastric carcinogenesis. The virulence factors CagA and VacA converge on host PTM systems rather than on any single pathway: translocated CagA is tyrosine-phosphorylated at EPIYA motifs to engage SHP2 and downstream proliferative signaling, and it drives the ubiquitin-dependent degradation of the tumor suppressors p53 (through AKT1-mediated HDM2 activation) and p14ARF, allowing DNA-damaged cells to survive [40]. VacA, in turn, disrupts autophagy and thereby impairs the clearance of ubiquitinated substrates and of CagA itself. These examples illustrate that the same writers, erasers, and ligases that constitute the crosstalk network can be co-opted by an oncogenic pathogen to shift the balance of key switches during early carcinogenesis, reinforcing the view that the network, rather than any single enzyme, is the relevant unit of dysregulation in gastric cancer.

## 4. PTM Crosstalk Across Cancer Hallmarks in Gastric Cancer

The crosstalk axes are not confined to individual substrates but converge on a set of malignant phenotypes that collectively determine the aggressiveness of gastric cancer (Figure 4). The four main manifestations directly caused by PTM crosstalk are sustained proliferation, invasion and metastasis, therapeutic resistance and immune evasion. These four phenotypes are not chosen arbitrarily; they represent the clinically relevant cancer hallmarks most profoundly shaped by PTM crosstalk in gastric cancer [41]. These phenotypes share the same underlying logic: the balance between competing and cooperative modifications determines whether a tumour-suppressing signal is reinforced or an oncogenic programme is unleashed, and it is through these phenotypic readouts that crosstalk ultimately translates into patient outcomes. As a result of this organisation, a single crosstalk switch can contribute to more than one hallmark—for example, SENP1 simultaneously regulates metabolic reprogramming and immune evasion—and such switches therefore represent rational therapeutic targets. The following subsections examine the specific mechanisms and therapeutic implications of PTM crosstalk in each of these four hallmarks.

### 4.1. Proliferation

Unrestricted proliferation is most directly coupled to the ubiquitination–deubiquitination balance: E3 ligases such as TRIM11 and RINES drive proliferation by degrading the tumor suppressor Axin1 and derepressing STAT3/MYC, respectively, whereas deubiquitinases such as USP48, JOSD1, and FAM105A stabilize oncogenic effectors to sustain the PI3K/AKT, Wnt7B-FZD1, and Hippo/YAP pathways [5,6,43,44,45]. The shared mechanism underlying these examples is the E3 ligase–deubiquitinase balance, whose state determines the strength of proliferative signaling. Because a single deubiquitinase can stabilize several effectors and a single E3 ligase can degrade several suppressors, dysregulation of one enzyme propagates across parallel proliferative programs. This convergence makes switch enzymes attractive targets, but it also cautions that their inhibition may affect normal proliferating tissues, a consideration that returns in the therapeutic discussion.

### 4.2. Invasion and Metastasis

Invasion and metastasis are the phenotypic manifestations of crosstalk across the proteome, epigenome, and metabolic state of cells. As described in Section 3.3, both lactylation and delactylation promote metastasis (S100A11 lactylation and LDHB delactylation), while histone lactylation at H3K18 and the HOXD9 promoter drives metastasis through transcriptional regulation [12,37,38,39]. In parallel, crosstalk is transmitted by microenvironmental relays, as shown by the hypoxia-induced EPAS1/HIF2A–USP33–ATG101 axis that promotes oncogenic autophagy and peritoneal metastasis [46]. Similarly, the ubiquitin system targets the EMT master regulator Snail: three deubiquitinases—USP35 [47], USP13 [48], and USP29 [49]—stabilize Snail and promote invasion and metastasis, whereas USP10 acts oppositely by stabilizing TNFRSF10B to restrain EMT [50]; notably, USP29 cooperates with the phosphatase SCP1, coupling phosphorylation status to Snail stability, although whether the other deubiquitinases are similarly regulated by upstream modifications remains to be established. Additionally, kinase and epigenetic enzymes contribute in parallel: the EphA2–YES1–ANXA2 phosphorylation pathway [51] and the histone demethylase PHF8 via the PKCα–Src–PTEN axis [52] both drive metastasis, although whether their downstream effects converge on ubiquitination remains to be clarified. Collectively, metastasis is driven by coordinated crosstalk among phosphorylation, ubiquitination, lactylation, and epigenetic modifications. Because the individual steps—detachment, invasion, intravasation, and colonization—each depend on distinct switches, the metastatic cascade can be viewed as a sequence in which different crosstalk events become rate-limiting at successive stages, so interventions that block one step may be effective only in the corresponding phase.

### 4.3. Therapeutic Resistance

Therapeutic resistance is a hallmark in which crosstalk provides both the mechanism of escape and, in some cases, a predictive marker. The SUMOylation–ubiquitination and phosphorylation–ubiquitination relays drive resistance to conventional agents: SENP1/ENO1 [7] and TRIM28/PCBP2 [53] promote cisplatin and radioresistance, respectively, whereas WNK1/PHGDH [54] and CK1δ/ATF4 [29] contribute to oxaliplatin sensitivity and chemoresistance. Deubiquitinases further regulate drug sensitivity through distinct downstream effectors: USP5 promotes chemoresistance by destabilizing p53 [55], and ferroptosis resistance emerges as a common theme, in which USP29 cooperates with SMURF1 to suppress FSP1-mediated ferroptosis [56] while the p53–OTUD5–GPX4 axis controls ferroptosis sensitivity [57]; whether the activities of these deubiquitinases are themselves regulated by upstream phosphorylation or acetylation remains largely unestablished. Whether the metabolic arm contributes to resistance through lactate-driven modifications also remains to be clarified. These findings indicate that resistance is often encoded not by an acquired mutation but by a shift in the state of a crosstalk switch, and such switch states may serve as biomarkers—for example, high SENP1 expression correlates with poor prognosis in gastric cancer [58]—that can be read out and manipulated to restore drug sensitivity. Resistance is thus a dynamic and potentially reversible property of the modification network: the same switch may contribute to resistance against multiple agents, and a single resistance phenotype may reflect the combined dysregulation of several switches.

### 4.4. Immune Evasion

Immune evasion links crosstalk with the tumour microenvironment, and these mechanisms are employed by multiple cell types rather than by a single cell. The ubiquitin arm directly suppresses anti-tumor immunity: the E3 ligase MDM2 impairs CD8+ T-cell function by degrading somatostatin [4], and PD-L1 abundance is regulated by deubiquitinases such as USP7 [42]—although whether USP7-mediated deubiquitination of PD-L1 is itself regulated by upstream phosphorylation or acetylation remains to be clarified—and by TRIM28-mediated stabilization [33], making PD-L1 stability a critical crosstalk-regulated node in immune evasion. The metabolic arm operates in both the tumour and immune compartments: lactate-driven histone lactylation in dendritic cells promotes PDIA6-mediated immune evasion [59], lactylation orchestrates immune evasion through KDM5B-mediated histone demethylation [13], and cancer-associated fibroblast-derived lactate suppresses NCAPG ubiquitination by means of histone lactylation to further drive immune evasion [60]. Thus, crosstalk links the metabolic state of the tumour to the suppression of anti-tumour immunity, joining metabolic reprogramming and immune escape into one regulatory continuum, and suggests that lactylation may represent a promising intervention node, as supported by pharmacological inhibition of histone lactylation enhancing immunotherapy [61]. Immune evasion differs from the other three hallmarks in that it operates across cell types: the same modifications act in tumour cells, dendritic cells, and cancer-associated fibroblasts, so the crosstalk events that shape immunity cannot be understood from tumour-cell-intrinsic mechanisms alone. This intercellular dimension adds complexity, but also opportunity, because the tumour microenvironment itself may be reprogrammed by targeting the shared metabolic or ubiquitin systems that drive immune escape. The same logic applies to proliferation, metastasis, resistance and immune evasion (Table 1): a single switch can promote multiple hallmarks, as exemplified by SENP1, so targeting one switch enzyme may affect several hallmarks simultaneously—an advantage for monotherapy that must nevertheless be weighed against context-dependent and potentially opposing effects. Most crosstalk-to-phenotype links have nonetheless been established for single proteins, and how the network-level state of crosstalk maps onto clinical behaviour and patient outcome is still largely unknown; these questions motivate the comparison of gastric and colorectal cancers in the following sections. The preceding analysis supports one central claim: the oncogenic output of gastric cancer is a network property, in which the balance among competing and cooperating modifications—not any single mark—determines protein fate and pathway activity, so single-modification measurements are inherently incomplete and therapeutic targeting should be redirected from individual marks toward the switches that set their balance.

## 5. Shared Principles and Divergent Features in Colorectal Cancer

Comparing gastric and colorectal cancer is instructive because these two common gastrointestinal malignancies share the same crosstalk framework yet differ in their driver mutation landscapes, microenvironments, and metabolic dependencies. Such a comparison reveals which features of the PTM network are general principles of gastrointestinal malignancy—and therefore potentially pan-gastrointestinal therapeutic targets—and which are cancer-specific switches that must be validated separately (Figure 5, a Venn diagram of shared principles and divergent switches). The shared ground lies at the level of the conceptual framework rather than in scattered individual examples: both cancers use competition, cooperation, and metabolic coupling to set the balance of modification that determines protein fate, and both converge on the same hallmark readouts of proliferation, metastasis, resistance, and immune evasion. The divergence, in turn, reflects which specific switches each tumour type preferentially deploys—a preference likely shaped by their distinct driver mutation landscapes and tissue microenvironments—and therefore how biomarkers and targets should be translated between the two diseases. The following subsections compare the specific switches shared or divergent between the two cancers and their translational implications.

### 5.1. Shared Principles

Three crosstalk modes are shared between the two cancers. First, metabolic coupling through lactylation links glycolytic metabolism to oncogenic and microenvironmental programs in both cancers: as in gastric cancer (Section 3.3), histone lactylation promotes colorectal cancer progression through circRNA-facilitated FXR1 phase separation—a liquid–liquid phase separation process that assembles membraneless organelles involved in gene regulation [65], and lactylation in cancer-associated fibroblasts induces extracellular matrix remodeling and immunosuppression [66], mirroring the CAF-derived lactate-driven immune evasion seen in gastric cancer (Section 4.4). Second, the phosphorylation–ubiquitination relay operates in both cancers, converting phosphorylation into ubiquitin-dependent degradation through CK1δ–ATF4 in gastric cancer (Section 3.1) and CDK1–ACSL4 in colorectal cancer, which is detailed in Section 5.2 [62]. By contrast, clear SUMO–ubiquitin competition has so far been documented mainly in gastric cancer (Section 3.2), and whether the SUMOylation–ubiquitination interplay is equally operative in colorectal cancer remains to be established.

### 5.2. Cancer-Specific Switches

Despite these shared principles, colorectal cancer also deploys switches with distinct substrates and functional outcomes, which can be grouped by modification type.

The phosphorylation–ubiquitination relay on ACSL4 is the most complete crosstalk example. CDK1-mediated phosphorylation recruits the E3 ligase UBR5 to promote ACSL4 degradation, thereby suppressing ferroptosis and conferring oxaliplatin resistance [62]; ACSL4 is a key ferroptosis regulator, and its control is especially relevant to colorectal cancer given the distinct lipid metabolism of this tumour type.

E3 ligases also control the Wnt/β-catenin pathway, which is hyperactive in colorectal cancer: NEURL promotes β-catenin degradation independently of GSK3β and β-TrCP [67], and BTBD3 ubiquitinates β-catenin to suppress tumourigenesis [68]; whether the activities of these E3 ligases are themselves regulated by upstream phosphorylation remains to be clarified.

Lactylation acquires colorectal-specific functions: PKM2 lactylation promotes vasculogenic mimicry and bevacizumab resistance by facilitating FOSL1 super-enhancer formation [69], and in KRASG12C-mutant liver metastasis, AKR1B10-driven histone lactylation reprograms neutrophils toward an immunosuppressive state that fosters immune evasion [70].

A further repertoire of single-modification regulators—deubiquitinases (USP9X, USP43, USP7, USP28, USP47, USP5, OTUB1) and SUMOylation enzymes (UBC9, SENP1, SENP3, UHRF2)—act on distinct substrates; although these are single-modification events rather than crosstalk, their cancer-specific substrates illustrate how the same enzyme class can be either oncogenic or tumour-suppressive depending on which substrate it protects [63,64,71,72,73,74,75,76,77,78,79].

These switches route the crosstalk logic through substrates tied to colorectal cancer biology: ferroptosis, vasculogenic mimicry, and the colorectal immune microenvironment. One generalization emerges from this comparison. The three types of PTM crosstalk, namely competition, cooperation and metabolic coupling, are conserved in both gastric and colorectal cancers, and may be general features of the modification network in gastrointestinal tumours. The only difference is that the switches and the paths they control are different; thus, the drivers, microenvironments and metabolic dependencies of the two diseases are also distinct. The biological basis of this divergence likely lies in the distinct tissue contexts: the two organs differ in their stem-cell architecture, microbiota exposure, and metabolic environment, and these differences plausibly determine which substrates are most vulnerable to a given crosstalk event and which switches are therefore most oncogenically relevant. Therefore, mechanistically derived knowledge is transferable at the level of concepts; that is, a switch defined in one type of cancer can often be explored in another, but therapeutic targets and biomarkers should not be assumed to be the same across different cancers and must be verified specifically in those cancers. Based on the above comparison, three key questions arise. First, which shared switches are genuine pan-gastrointestinal dependencies and which are merely recurrent? Second, to what extent do the divergent switches reflect differences in cell origin, microbiota, or metabolic environment? Third, can these determinants be used to predict which tumours will rely on a particular crosstalk axis? Resolving these questions would provide a path for converting crosstalk biology into therapy and would set up the therapeutic strategies discussed in the following section. The comparative approach also carries a methodological lesson: because the two cancers share a conceptual framework while differing in its concrete instantiations, cross-cancer validation offers a natural test of whether a given switch is a general dependency or a context-specific one—a distinction that cannot be resolved by studying either cancer in isolation.

### 5.3. An Unbiased Transcriptome-Level Comparison

To test the comparative framework in an unbiased manner, rather than through curated examples alone, we retrieved transcriptome-wide expression data for gastric and colorectal adenocarcinoma from the TCGA PanCancer Atlas (RNA-seq V2 RSEM) and identified genes whose expression in primary tumors deviated significantly from normal tissue, using z-scores computed relative to normal samples. In a randomly selected subset of 40 primary tumors per cancer type, a gene was considered significantly dysregulated when its mean z-score across tumors exceeded |2| (Section Methods (Bioinformatic Comparison)). This identified 543 dysregulated genes in gastric cancer (380 up-regulated, 163 down-regulated) and 4636 in colorectal cancer (1815 up-regulated, 2821 down-regulated), of which 385 were shared and directionally concordant (372 concordant, 13 discordant). The larger dysregulated repertoire of colorectal cancer is consistent with its more heterogeneous molecular landscape, which includes hypermutated/microsatellite-instable and highly aneuploid subtypes that drive broader transcriptional remodeling [80].

The shared set of 385 genes defines a conserved, cancer-type-independent core. STRING protein–protein interaction (PPI) analysis of this set (confidence score ≥ 0.4) recovered a densely connected network of 1,673 edges, whose highest-degree hubs were the ribosome-biogenesis factors NOP56, GTPBP4, NOP58, and DKC1, the chaperones HSP90AA1/HSP90AB1, and the DNA-repair regulator BRCA1 (Figure 6). Consistent with this topology, KEGG and Gene Ontology (biological process) enrichment of the shared set was dominated by nucleic acid metabolism, rRNA processing and ribosome biogenesis, DNA replication, and ribonucleoprotein-complex biogenesis (all FDR < 0.05). The prominence of HSP90 is notable in the context of PTM crosstalk, because HSP90 is itself a hub whose client stability is governed by ubiquitination, phosphorylation, and acetylation, and whose inhibition triggers proteasomal clearance of oncogenic clients—a network property that recapitulates the “switch-enzyme” logic developed in Section 3 and Section 6 [41].

By contrast, the cancer-type-specific sets point to distinct regulatory vulnerabilities. Gastric-cancer-specific genes were enriched for epigenetic regulators, with DNMT1, NSUN2, and SRSF1 among the top network hubs, suggesting that DNA and RNA methylation—both intimately coupled to the ubiquitin system through reader and eraser enzymes—represent a gastric-biased layer of the crosstalk network. Colorectal-cancer-specific genes were instead dominated by colon-lineage markers whose down-regulation reflects loss of differentiation (GUCA2A/GUCA2B, CLCA1) together with the cAMP-dependent kinase PRKACB and immune/stromal mediators (IL4, BCL2), echoing the distinct Wnt-driven and immune-active biology of colorectal tumors described in Section 5.2. These unbiased results therefore converge with the curated analysis: the three crosstalk modes and their hallmark readouts are conserved across the two cancers, whereas the concrete switches—the epigenetic and lineage-specific substrates through which the network is wired—diverge, exactly as the comparative framework predicts.

#### Methods (Bioinformatic Comparison)

TCGA PanCancer Atlas mRNA expression (RNA-seq V2 RSEM, batch-normalized) for stomach adenocarcinoma (STAD, *n* = 440 tumors) and colorectal adenocarcinoma (COADREAD, *n* = 594 tumors) was accessed through the cBioPortal API. Per-gene expression *z*-scores relative to normal tissue were retrieved for a randomly selected subset of 40 primary tumors per cancer type (fixed random seed), and a gene was classified as significantly dysregulated when its mean z-score across tumors exceeded |2|. Gene sets (shared, gastric-specific, and colorectal-specific) were compared; protein–protein interaction networks were constructed with STRING v12 (confidence score ≥ 0.4) for the shared set, and functional enrichment (KEGG and Gene Ontology biological process) was performed with the STRING enrichment tool, with significance defined as FDR < 0.05. Analyses used Python 3.10 (networkx 3.4.2, SciPy 1.15.3); Figure 6 was generated with matplotlib 3.10.9. No statistical assumption was made about the direction of dysregulation, and the analysis is descriptive and hypothesis-generating rather than confirmatory.

**Figure 6:**
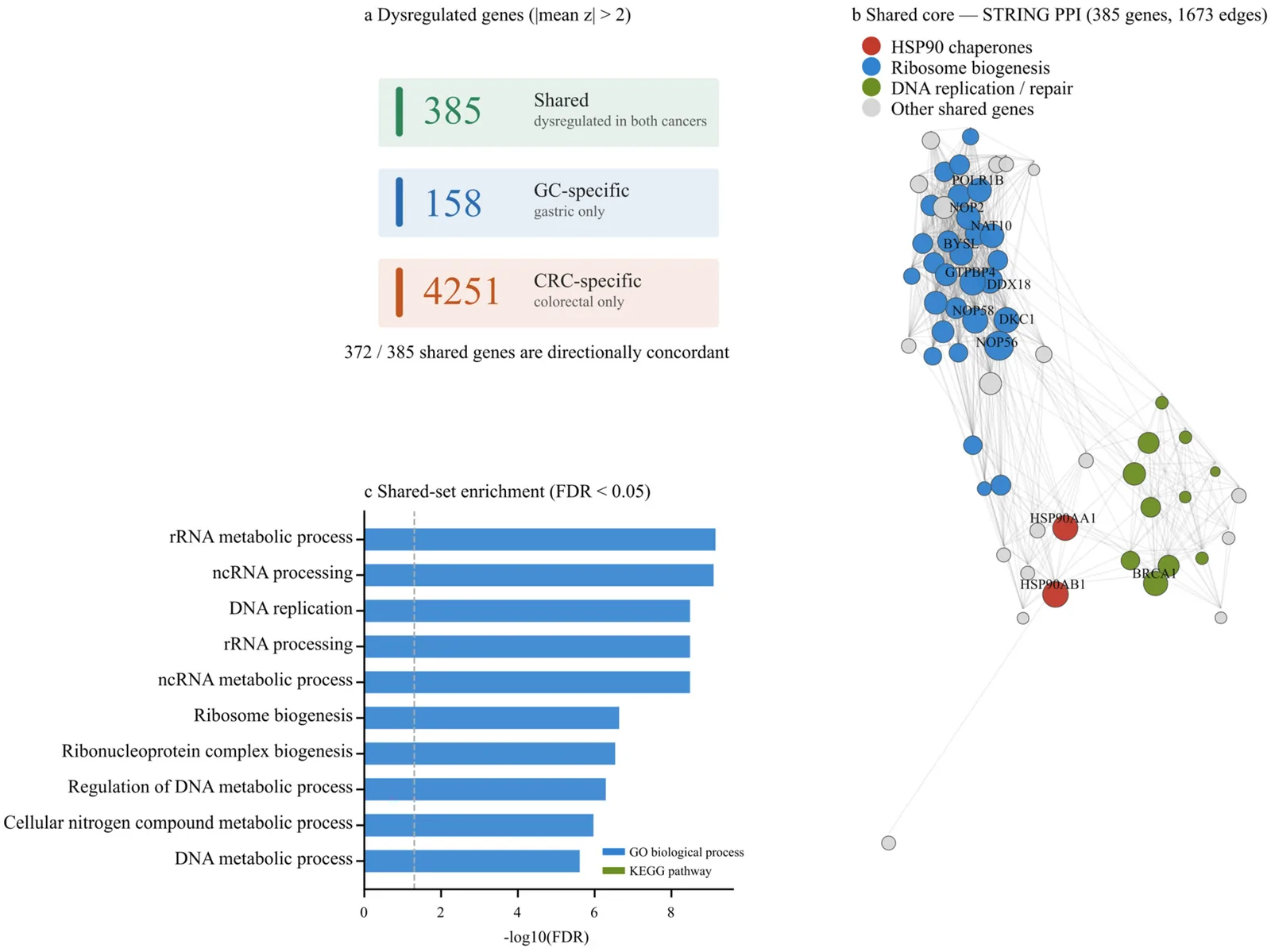
Unbiased transcriptome-level comparison of gastric and colorectal cancer. Bioinformatic comparison of significantly dysregulated genes in gastric cancer (GC, *n* = 543) and colorectal cancer (CRC, *n* = 4636), identified from TCGA PanCancer Atlas mRNA z-scores relative to normal tissue (|mean z| > 2 in a random subset of 40 primary tumors per cancer). (**a**) Counts of shared (*n* = 385) and cancer-specific dysregulated genes; 372 of 385 shared genes are directionally concordant. (**b**) STRING protein–protein interaction network of the 385 shared genes (confidence ≥ 0.4; 1673 edges), colored by function and with the highest-degree hubs labeled—ribosome biogenesis factors (NOP56, GTPBP4, NOP58, DKC1), the HSP90 chaperones (HSP90AA1/HSP90AB1), and the DNA-repair regulator BRCA1. (**c**) KEGG and Gene Ontology (biological process) enrichment of the shared set (FDR < 0.05), dominated by ribosome biogenesis, rRNA processing, and DNA replication.

## 6. Therapeutic Targeting of PTM Crosstalk

The crosstalk-centered view of gastric cancer has a direct therapeutic implication (Table 2): if oncogenic output is regulated by the balance of competing and cooperating modifications, then the most informative targets are not single modifications but “switch” enzymes that alter the relative activities of these modifications. This view offers a new perspective for drug discovery, steering it from a residue-by-residue catalogue of marks toward the writers, erasers, and ligases that add, subtract, and modify them. Because the three crosstalk modes map onto different targeting logics, the therapeutic landscape can be organised around the same framework used to understand the biology. The targeting logic follows from the mode of crosstalk. For competitive toggles, in which modifications vie for the same residue, the aim is to alter the balance by inhibiting the dominant writer, activating the opposing eraser, or blocking the stabilizing kinase—for instance, when phosphorylation promotes SUMOylation over ubiquitination, blocking that kinase would restore ubiquitination-mediated degradation. For cooperative cascades, in which one modification instructs another, the most efficient intervention is to block the recognition step, for example by preventing a phosphodegron from recruiting its E3 ligase, although disrupting such protein–protein interactions remains a recognized challenge. For metabolic coupling, the target can be the metabolite itself, the ligase that installs the modification, or the eraser that removes it, and targeting the modifying enzymes may prove more selective than targeting the metabolite itself. The same logic applies to gastric and colorectal cancer alike, and the following subsections discuss specific targets and agents for each logic.

### 6.1. Targeting the Ubiquitin System

The deubiquitinase and E3 ligase arms of the ubiquitin system offer the clearest illustration. Deubiquitinases that stabilize oncogenic effectors are attractive targets, and their inhibition releases the degradative arm. USP7 has been pursued as such a target: a homologous cell-membrane-coated peptide-based USP7 degrader—which induces target-protein degradation, in contrast to inhibitors that merely block enzyme activity—enhances degradation efficiency and antitumor activity in gastric cancer [81], although selective USP7 inhibitors have not yet entered clinical trials [82], and the selectivity, off-target effects, and pharmacokinetic properties of this degrader remain to be characterized beyond preclinical models. Beyond directly targeting oncogenic proteins, the ubiquitin system also governs the stability of immune checkpoints, thereby connecting ubiquitin targeting to immunotherapy. PD-L1 is regulated by deubiquitinases such as USP32 [83] and USP33 [84] in colorectal cancer, and modulating PD-1/PD-L1 ubiquitination—typically to promote PD-L1 degradation and relieve immune suppression—is emerging as a strategy to overcome immunotherapy resistance [85]; whether the regulatory networks of USP32 and USP33 involve multi-modification crosstalk remains to be clarified. These colorectal findings suggest that ubiquitin-system regulation of tumour immunity is general across gastrointestinal cancers, although the specific targets may differ between cancer types. The E3 side offers a complementary opportunity: inhibiting ligases that degrade tumour suppressors or immune factors could restore the restraining effect, although the human genome encodes more than 600 E3 ligases, many with multiple substrates, so achieving selective inhibition remains a major technical hurdle. This duality—E3 ligases and deubiquitinases as opposing but equally druggable arms—is a recurring theme across the ubiquitin system. The most effective intervention may not always be the most obvious: in some cases restoring an endogenous degradation event, rather than blocking a stabilizing enzyme, may be the more selective route to the same therapeutic end.

### 6.2. Targeting SUMOylation and deSUMOylation

The SUMOylation–deSUMOylation axis provides a second class of targets, centered on the SENP deSUMOylases. SENP1 is the best-validated example in gastric cancer: it deSUMOylates ENO1 at K256 and K394 to maintain glycolysis and confer cisplatin resistance [7], and disruption of the SUMOylation-mediated SREBP2–FBXW7 interaction promotes de novo cholesterol synthesis [34]; the SENP1 inhibitor Momordin Ic in combination with cisplatin shows a synergistic effect on gastric tumor growth in vitro and in vivo [7], suggesting that SENP1 inhibition may offer dual benefits against metabolic dependence and chemoresistance, although direct evidence linking both effects in a single model remains to be accumulated. The corresponding SUMO E3 enzymes are also relevant but have lagged behind as drug targets, in part because SUMOylation proceeds through an E1–E2–E3 cascade in which UBC9 is the sole SUMO E2, and the conserved active sites of these enzymes limit selective targeting. The recurrence of SENP1 across metabolic, resistance, and lipid programmes of gastric cancer suggests that deSUMOylases may be more readily targeted and broadly effective than individual SUMO substrates; however, SENP1 has multiple substrates, some of which may be tumour-suppressive, so its inhibition carries potential off-target effects that must be systematically evaluated before translation. Although other SENP family members, such as SENP2 and SENP3, are also reported in gastric cancer, SENP1 remains the most studied target owing to its multiple roles in metabolism and resistance. The broader lesson from SENP1 is that a single deSUMOylase can act as a hub for several oncogenic programs at once, so its inhibition offers a form of network-level intervention that differs in kind from targeting any individual SUMO substrate. This hub property is a double-edged sword, however, and it reinforces the need for careful selectivity and safety evaluation, a theme that recurs for every switch enzyme discussed here.

### 6.3. Targeting Lactylation and Metabolic Coupling

The metabolic coupling arm is the most recent addition to the therapeutic landscape, and lactylation is its principal target. Lactylation enzymes are under active investigation as new therapeutic targets [86]: the writers include the MYST-family acetyltransferases (such as KAT2A) and the metabolic sensors AARS1/2 [86], and acetyltransferase inhibitors such as A-485 have been reported to affect lactylation levels, although gastric-cancer-specific evidence remains to be established. Together with lactate metabolism, these enzymes define a pathway from pathogenesis to therapy [87]. Disrupting the glycolysis–lactylation axis may therefore uncouple the Warburg effect from its immunosuppressive and resistance-promoting consequences [88], an approach that is especially appealing in gastric and colorectal cancer, where this axis is repeatedly implicated. Consistent with this, targeting lactylation-related pathways—for example, through lactyltransferase inhibitors or lactate metabolism intervention—has been proposed to enhance immunotherapy efficacy in gastrointestinal cancers by restoring antitumor immunity [61]. Lactylation of PD-L1 at K280 likewise stabilizes the checkpoint to promote immune evasion [36]. Because lactylation is dynamic—installed by lactyltransferases and removed by erasers—it offers the same dual-sided control as the ubiquitin and SUMO systems; agents that intervene on lactate metabolism, such as LDHA inhibitors (e.g., GNE-140) or metformin, have been used to target the lactate pool, although their specific effects on lactylation remain to be validated. The identity of the specific delactylases and the selectivity of candidate inhibitors also remain important open questions. The translation of these three targeting logics nonetheless faces mode-specific challenges. For competitive toggles, selectivity is the central obstacle. Because switch enzymes act on multiple substrates across oncogenic and homeostatic processes, pan-inhibition risks pleiotropic toxicity, and degraders such as PROTACs, while more potent, do not by themselves solve selectivity; achieving cancer-specific targeting will require substrate-selective agents or allosteric modulators that bias a switch rather than abolish it. For cooperative cascades, the key unmet need is predictive biomarkers: identifying which tumours depend on a given relay would enable patient selection, yet such biomarkers remain largely uncharacterized. For metabolic coupling, the bottleneck is the lack of systematic, proteome-scale maps of crosstalk that would guide target prioritization across the modification network. Progress on these fronts—substrate-selective switch modulators, crosstalk-aware biomarker discovery, and multi-omics profiling—would convert the crosstalk framework from a conceptual scaffold into actionable therapies for gastric and colorectal cancer. A further practical consideration is that combination therapy may be the most realistic path forward. Because switches often converge on the same hallmarks, combining a switch-directed agent with existing chemotherapy, targeted therapy, or immune checkpoint blockade could restore sensitivity without requiring the new agent to be potent as a monotherapy—a rationale already illustrated by the synergy between the SENP1 inhibitor Momordin Ic and cisplatin.

## 7. Conclusions

The oncogenic effect of gastric cancer is not primarily due to single post-translational modifications, but rather to crosstalk networks that coordinate competitive, cooperative and metabolic changes among these modifications to regulate protein fate and pathway activities. Phosphorylation–ubiquitination relays, SUMO-ubiquitin interplay and lactate-driven lactylation all employ the same logic across substrates and hallmarks, and are also present in gastric and colorectal cancers. This framework has direct practical applications: single-modification studies often yield conflicting results because the functional state of a protein is determined by the balance of modifications, not by any single modification, so experimental design should be oriented towards a network rather than a single mark, and the switch enzymes—competing writers, erasers and ligases—emerge as highly informative therapeutic targets and potential biomarkers. For example, high SENP1 expression correlates with poor prognosis in gastric cancer and thus offers a candidate biomarker for patient stratification [58], while preclinical data with a USP7 degrader provide proof-of-concept for targeting switch enzymes [81]. Several limitations of the current evidence base should nonetheless be acknowledged. Many of the mechanisms discussed derive from single cell-line models or xenografts rather than patient-derived or in vivo validation, and sample sizes in individual studies are often modest; the field would benefit from independent replication and from orthogonal, patient-derived systems. In addition, most studies report a single modification on a single substrate, so the network-level picture synthesized here necessarily integrates findings across different experimental systems rather than measuring crosstalk directly in one model, and contradictory findings across studies remain to be reconciled. Finally, the therapeutic candidates highlighted are at preclinical stages, and their translation will depend on resolving selectivity, pharmacokinetics, and off-target toxicity, none of which are yet established. These caveats temper, but do not undermine, the central claim that the crosstalk network is the relevant unit of dysregulation in gastric cancer.

## 8. Future Perspectives

Realizing this value faces the following challenges: current experimental models seldom capture the dynamics and spatial regulation of modification networks; multi-modification omics data are difficult to integrate and standardise; and the field needs to incorporate chemical proteomics and single-cell or spatial approaches—spatial information matters because the intracellular distribution of modifying enzymes and their substrates is critical for the spatiotemporal specificity of signalling—increasingly empowered by artificial intelligence (for example, for integrating multi-omics data, predicting modification sites and their crosstalk events, and building network-level regulatory models), to map and predict crosstalk at scale. Although the integration of these technologies is still at an early stage, systematic maps of crosstalk networks may become feasible as single-cell multi-omics and AI prediction tools mature. Resolving these technical problems would accelerate the translation of switch-targeting therapies and biomarkers, and the resulting clinical and mechanistic data would in turn refine, extend, and test the crosstalk framework itself, advancing gastric and colorectal cancer research beyond a list of markers towards an all-encompassing understanding of modification networks. Importantly, this progress need not await a complete map of the network: even partial knowledge of a switch—its key substrates, its writers and erasers, and its functional readouts—is sufficient to motivate targeted experiments and early-stage clinical hypotheses. The field’s trajectory therefore runs from single-protein, single-modification studies, through the axis-by-axis synthesis attempted here, toward a quantitative, network-level understanding that is likely to reshape both the mechanistic and the therapeutic view of gastric cancer.

## Figures and Tables

**Figure 1:**
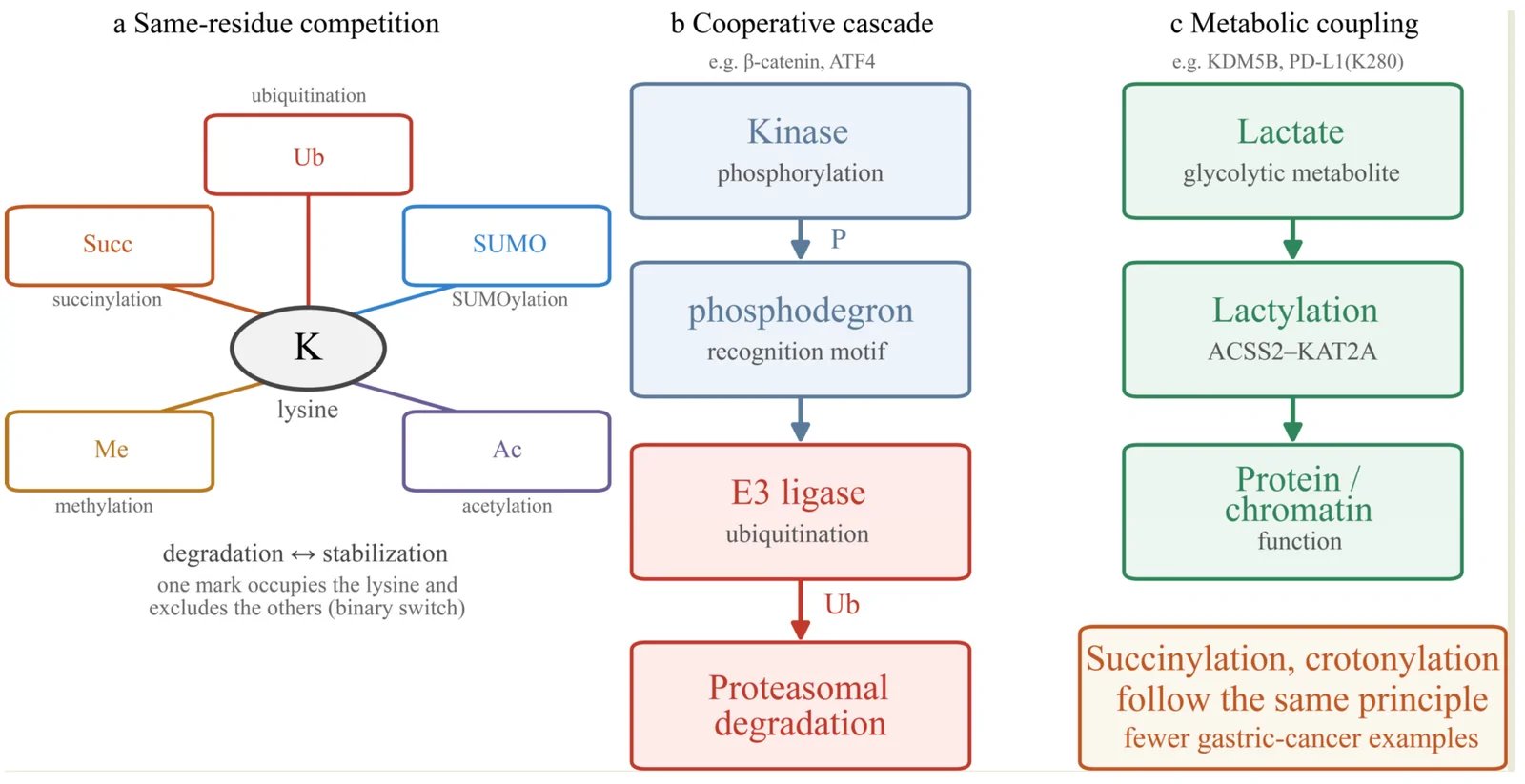
Three modes of PTM crosstalk. Schematic overview of the three principal modes of post-translational modification (PTM) crosstalk. (**a**) Same-residue competition, in which multiple lysine-directed modifications—ubiquitination, SUMOylation, acetylation, methylation, and succinylation—compete for the same lysine residue, so occupancy by one excludes the others and toggles protein fate (e.g., acetylation–ubiquitination on ATP-citrate lyase [14]; SUMO–ubiquitin competition on MYC K51/K52 by PIAS1 [15]). (**b**) Cooperative crosstalk across residues, in which one modification generates a recognition motif that instructs a second—exemplified by the phosphorylation–ubiquitination phosphodegron (β-catenin [16,17]) and acetylation-directed ubiquitination (PSAT1 [18]; TRIM25–USP7 [19]). (**c**) Metabolic/epigenetic coupling, in which a metabolite-derived modification links cellular metabolic state to protein or chromatin function; lactylation is singled out as the best-evidenced mark linking glycolysis to protein stability and immunity [20], whereas succinylation and crotonylation follow the same principle but have fewer gastric-cancer demonstrations (e.g., CPT1A-mediated succinylation of S100A10 [21]).

**Figure 2:**
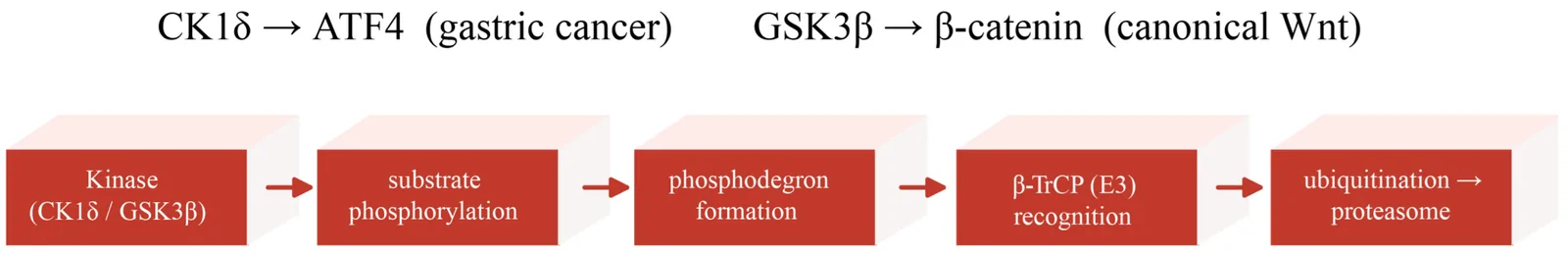
Phosphorylation–ubiquitination relays in gastric cancer. The phosphorylation–ubiquitination relay as a cooperative crosstalk axis. A kinase (CK1δ or GSK3β) phosphorylates a substrate (ATF4 or β-catenin) to generate a phosphodegron, which recruits the SCFβ-TrCP E3 ligase for ubiquitination and proteasomal degradation, converting kinase activity into protein turnover [16,17,29].

**Figure 3:**
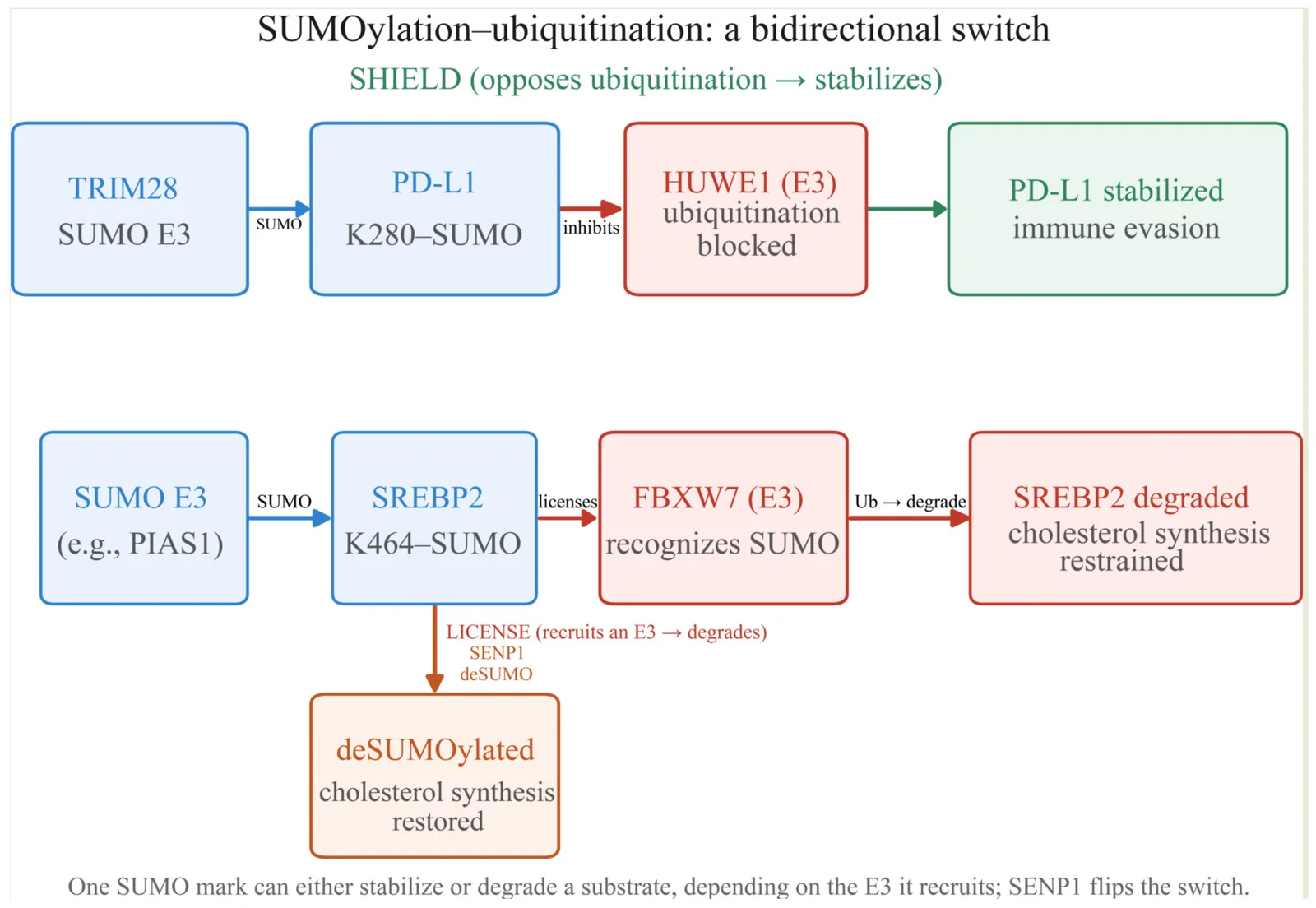
SUMOylation–ubiquitination bidirectional switch in gastric cancer. The SUMOylation–ubiquitination axis acts as a bidirectional switch in gastric cancer. In the shielding arm, TRIM28-mediated SUMOylation of PD-L1 at K280 blocks HUWE1-mediated ubiquitination, stabilizing the checkpoint to promote immune evasion [33]. In the licensing arm, SUMOylation of SREBP2 at K464 licenses recognition by the E3 ligase FBXW7 for ubiquitin-dependent degradation, and SENP1-mediated deSUMOylation disrupts this interaction to drive de novo cholesterol synthesis [34]. SENP1 likewise stabilizes HIF-1α and ENO1 to drive glycolysis and cisplatin resistance [35].

**Figure 4:**
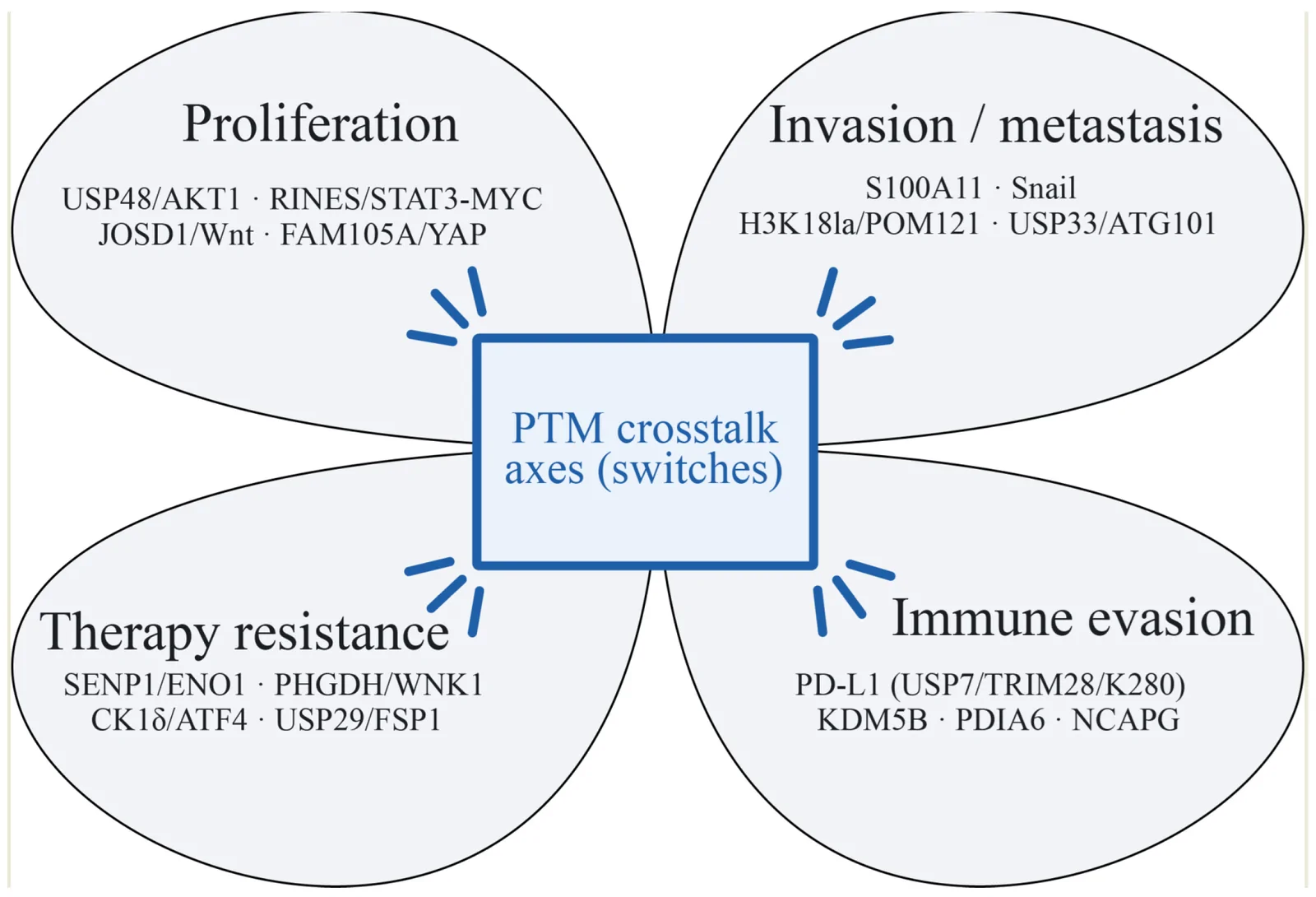
PTM crosstalk axes converge on cancer hallmarks. Mapping of crosstalk axes onto four malignant hallmarks in gastric cancer. Proliferation is driven by the E3–deubiquitinase balance (USP48/AKT1, RINES/STAT3-MYC, JOSD1/Wnt, FAM105A/YAP) [5]; invasion and metastasis by the Snail and lactylation axes (S100A11, Snail, H3K18 lactylation/POM121, USP33/ATG101) [12]; therapeutic resistance by the SENP1 and phosphodegron axes (SENP1/ENO1, PHGDH/WNK1, CK1δ/ATF4, USP29/FSP1) [7]; and immune evasion by the PD-L1 and lactylation axes (PD-L1 via USP7/TRIM28/K280, KDM5B, PDIA6, NCAPG) [42].

**Figure 5:**
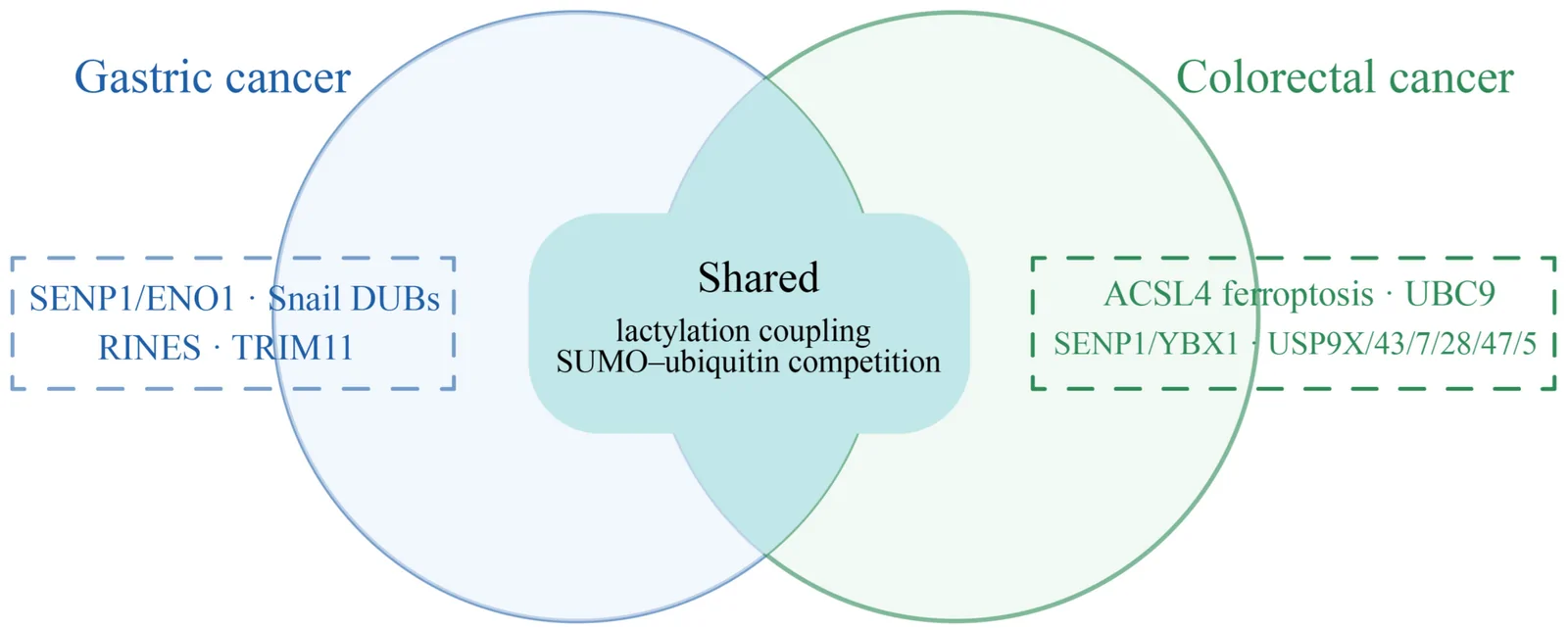
Shared principles and divergent features between gastric and colorectal cancer. Comparative view of PTM crosstalk in gastric versus colorectal cancer. Shared principles include metabolic coupling through lactylation and SUMOylation–ubiquitination competition. Gastric-cancer-specific features include the SENP1/ENO1 and Snail-stabilizing deubiquitinase axes, and the RINES/TRIM11 E3 ligases [7]. Colorectal-specific features include the ACSL4 phosphorylation–ubiquitination relay controlling ferroptosis [62], UBC9/SENP1–YBX1 SUMOylation [63], and a distinct repertoire of deubiquitinases (USP9X, USP43, USP7, USP28, USP47, USP5) [64].

**Table 1:** Representative PTM crosstalk switches in gastric and colorectal cancer.

Protein	Crosstalk	Key Enzymes	Functional Outcome
**β-catenin**	Phosphorylation → ubiquitination	GSK3β/β-TrCP	Degradation; Wnt restrained
**ATF4**	Phosphorylation → ubiquitination	CK1δ/β-TrCP	Degradation; chemoresistance
**ACSL4**	Phosphorylation → ubiquitination	CDK1/UBR5	Degradation; oxaliplatin resistance (CRC)
**PD-L1**	SUMOylation vs. ubiquitination	TRIM28	Stabilization; immune evasion
**SREBP2**	SUMOylation licences ubiquitination	SENP1/FBXW7	K464; degradation vs. stabilization
**ACLY**	Acetylation blocks ubiquitination	PCAF/SIRT2	K540/546/554; stabilization
**MYC**	SUMOylation vs. ubiquitination	PIAS1	K51/K52; stability control
**p14ARF**	N-terminal SUMOylation vs. ubiquitination	UBC9	Stabilization
**ABCG2**	Glycosylation vs. ubiquitination	—	Stabilization
**PD-1**	Deglycosylation → ubiquitination	MDM2/NGLY1	Degradation
**S100A11**	Lactylation shields degradation	—	K27/K55; metastasis
**PD-L1**	Lactylation blocks ubiquitination	AARS1/HUWE1	K280; stabilization
**cGAS**	Lactylation → ubiquitin-independent degradation	PSMA4	K21; degradation
**S100A10**	Succinylation blocks ubiquitination	CPT1A/SIRT5	K47; invasion
**LDHB**	Delactylation	HDAC	K58; ferroptosis resistance

**Table 2:** Therapeutic strategies targeting PTM crosstalk.

Target	Class	Strategy	Status
**USP7**	Deubiquitinase	Inhibitors/degraders	Preclinical (gastric degrader shown)
**USP32/USP33**	Deubiquitinase	PD-L1 axis	Preclinical (CRC)
**SENP1**	DeSUMOylase	Momordin Ic (with cisplatin)	Preclinical (gastric)
**Lactyltransferases (MYST/AARS)**	Lactylation writers	Inhibitors under development	Preclinical
**Histone lactylation**	Epigenetic	Targeting proposed to enhance immunotherapy	Proposed
**LDHA**	Metabolic enzyme	GNE-140/metformin	Clinical use; lactylation effects unvalidated

## Data Availability

No new data were created or analyzed in this study. Data sharing is not applicable to this article.

## References

[1] Bray F., Laversanne M., Sung H., Ferlay J., Siegel R.L., Soerjomataram I., Jemal A. (2024). Global cancer statistics 2022: GLOBOCAN estimates of incidence and mortality worldwide for 36 cancers in 185 countries. CA Cancer J. Clin..

[2] Peng N., Liu J., Hai S., Liu Y., Zhao H., Liu W. (2024). Role of Post-Translational Modifications in Colorectal Cancer Metastasis. Cancers.

[3] Hershko A., Ciechanover A. (1998). The ubiquitin system. Annu. Rev. Biochem..

[4] Yang F., Zhang X., Ji J., Li S., Fang X., Yang L., Guo X. (2026). E3 Ubiquitin Ligase MDM2 Promotes Growth and CD8+ T Cell Dysfunction in Gastric Cancer Through Degradation of SST. J. Gastroenterol. Hepatol..

[5] Zhao Y., Zhang Z., Xu A.M. (2025). HEG1 promotes gastric cancer progression by stabilizing AKT1 and is functionally regulated by the deubiquitinase USP48. Eur. J. Med. Res..

[6] Liu L., Ma Z., Zhang Y., Ye Z., Li H., Shi W., Jiao Z. (2026). JOSD1 stabilizes SULF1 to activate Wnt7B-FZD1 signaling in gastric cancer. Transl. Oncol..

[7] Fang Y., Gu Y., Xu T., Wang P., Wu X., Shen H., Xu Y., Xu Z., Cao L., Li X. (2025). SENP1 drives glycolysis and cisplatin resistance in gastric cancer via desumoylating ENO1. J. Exp. Clin. Cancer Res..

[8] Tabnak P., Ebrahimnezhad M. (2025). Insights into SUMOylation in gastric cancer: Molecular mechanisms and emerging therapeutic opportunities. J. Cancer Res. Clin. Oncol..

[9] Song H., Zhang M., Guo C., Guo X., Ma Y., Ma Y. (2025). Implication of protein post translational modifications in gastric cancer. Front. Cell Dev. Biol..

[10] Qiao K., Wu L., Yang L., Liu M., Jiang C., Chen Y., Zhang Z., Yu J., Wei D., Xu Y. (2026). Ubiquitin-centered post-translational modification crosstalk orchestrates tumor immunity and immunotherapy response. Exp. Hematol. Oncol..

[11] Fan X., Li Z., Gao Q., Huang P., Li Z., Lu D., Wang L., Xiang P., Huang T., Shen D. (2026). TRIM28 orchestrates SUMO-ubiquitin crosstalk to stabilize PPARG and drive bladder cancer progression. Cell Death Dis..

[12] Zhang Q., Zhou Y., Wang F., Yan J., Liu J., Zhang L., Du S. (2026). Lactylation shields S100A11 from degradation to promote gastric cancer metastasis. Cell. Oncol..

[13] Du W., Ding L., Zhu J., Li L., Wang X., Zhang Y., Wu X., Pei D. (2026). Lactylation Orchestrates Immune Evasion in Gastric Cancer through KDM5B-Mediated Histone Demethylation. Cancer Res..

[14] Lin R., Tao R., Gao X., Li T., Zhou X., Guan K.L., Xiong Y., Lei Q.Y. (2013). Acetylation stabilizes ATP-citrate lyase to promote lipid biosynthesis and tumor growth. Mol. Cell.

[15] Rabellino A., Melegari M., Tompkins V.S., Chen W., Van Ness B.G., Teruya-Feldstein J., Conacci-Sorrell M., Janz S., Scaglioni P.P. (2016). PIAS1 promotes lymphomagenesis through MYC upregulation. Cell Rep..

[16] Aberle H., Bauer A., Stappert J., Kispert A., Kemler R. (1997). beta-catenin is a target for the ubiquitin-proteasome pathway. EMBO J..

[17] Winston J.T., Strack P., Beer-Romero P., Chu C.Y., Elledge S.J., Harper J.W. (1999). The SCFβ-TRCP-ubiquitin ligase complex associates specifically with phosphorylated destruction motifs in IκBα and β-catenin and stimulates IκBα ubiquitination in vitro. Genes Dev..

[18] Liu Y., Xun W., Zhao T., Huang M., Sun L., Wen G., Kang X., Wang J., Han T. (2024). Interplay between acetylation and ubiquitination controls PSAT1 protein stability in lung adenocarcinoma. Commun. Biol..

[19] Yang J., Chen Z., Hu W., Zeng W., Lei Z., Tong X., Li Q., Luo G., Hu K., Chen Z. (2025). Acetylation-dependent USP7-TRIM25 axis drives oncogenic progression in non-small cell lung cancer. Cell Death Dis..

[20] Zhang D., Tang Z., Huang H., Zhou G., Cui C., Weng Y., Liu W., Kim S., Lee S., Perez-Neut M. (2019). Metabolic regulation of gene expression by histone lactylation. Nature.

[21] Wang C., Zhang C., Li X., Shen J., Xu Y., Shi H., Mu X., Pan J., Zhao T., Li M. (2019). CPT1A-mediated succinylation of S100A10 increases human gastric cancer invasion. J. Cell. Mol. Med..

[22] Chen Y., Sun X.X., Sears R.C., Dai M.S. (2019). Writing and erasing MYC ubiquitination and SUMOylation. Genes Dis..

[23] El Motiam A., Bouzaher Y.H., Chen H., Seoane R., Vidal S., Blanquer M., Tolosa R.M., Rodríguez-Lemus B., Herrera-Gavilán J.A., Vidal A. (2025). SUMOylation of the lysine-less tumor suppressor p14ARF counters ubiquitylation-dependent degradation. Cell Death Dis..

[24] Nakagawa H., Wakabayashi-Nakao K., Tamura A., Toyoda Y., Koshiba S., Ishikawa T. (2009). Disruption of N-linked glycosylation enhances ubiquitin-mediated proteasomal degradation of the human ATP-binding cassette transporter ABCG2. FEBS J..

[25] Wu Z., Cao Z., Yao H., Yan X., Xu W., Zhang M., Jiao Z., Zhang Z., Chen J., Liu Y. (2023). Coupled deglycosylation-ubiquitination cascade in regulating PD-1 degradation by MDM2. Cell Rep..

[26] Lei A., Chen L., Zhang M., Yang X., Xu L., Cao N., Zhang Z., Cao Y. (2019). EZH2 Regulates Protein Stability via Recruiting USP7 to Mediate Neuronal Gene Expression in Cancer Cells. Front. Genet..

[27] Lee J.Y., Park J.H., Choi H.J., Won H.Y., Joo H.S., Shin D.H., Park M.K., Han B., Kim K.P., Lee T.J. (2017). LSD1 demethylates HIF1α to inhibit hydroxylation and ubiquitin-mediated degradation in tumor angiogenesis. Oncogene.

[28] Kim H.J., Kim S.Y., Kim D.H., Park J.S., Jeong S.H., Choi Y.W., Kim C.H. (2020). Crosstalk between HSPA5 arginylation and sequential ubiquitination leads to AKT degradation through autophagy flux. Autophagy.

[29] Feng L., Li M., Hu X., Li Y., Zhu L., Chen M., Wei Q., Xu W., Zhou Q., Wang W. (2021). CK1δ stimulates ubiquitination-dependent proteasomal degradation of ATF4 to promote chemoresistance in gastric Cancer. Clin. Transl. Med..

[30] Zhu R., Ye X., Lu X., Xiao L., Yuan M., Zhao H., Guo D., Meng Y., Han H., Luo S. (2025). ACSS2 acts as a lactyl-CoA synthetase and couples KAT2A to function as a lactyltransferase for histone lactylation and tumor immune evasion. Cell Metab..

[31] Rao K., Zhang X., Luo Y., Xia Q., Jin Y., He J. (2025). Lactylation orchestrates ubiquitin-independent degradation of cGAS and promotes tumor growth. Cell Rep..

[32] Celen A.B., Sahin U. (2020). Sumoylation on its 25th anniversary: Mechanisms, pathology, and emerging concepts. FEBS J..

[33] Ma X., Jia S., Wang G., Liang M., Guo T., Du H., Li S., Li X., Huangfu L., Guo J. (2023). TRIM28 promotes the escape of gastric cancer cells from immune surveillance by increasing PD-L1 abundance. Signal Transduct. Target. Ther..

[34] Yang C., Wang Q., Han B., Shangguan X. (2026). SENP1 drives de novo cholesterogenesis via disrupting SUMOylation-mediated SREBP2-FBXW7 interaction. Cancer Metab..

[35] Zhu M., Liang B., Yang C., Jia J. (2026). SENP1 drives glycolytic reprogramming to promote malignant progression of gastric cancer via deSUMOylation of HIF-1α. Tissue Cell.

[36] Liang L., Zong Y., Huang J., Chen Y., Xu N., Yan P., Song H., Wu M. (2026). Lactylation stabilizes PD-L1 to promote tumor immune evasion and cell growth. Cell Death Dis..

[37] Wang E., Jia Y., Shen L., Guo K., Guo Z., Zhang H. (2026). H3K18 lactylation promotes POM121 transcription and accelerates gastric cancer progression via the PI3K/AKT pathway. Pathol. Res. Pract..

[38] Zhang H., Wang X., Sun N., Wang Z., Zhao C., Yin S., Zeng L., Zhang Y., Fu Y. (2026). CSDE1-mediated histone lactylation modification of the HOXD9 promoter promotes gastric cancer progression. Biochem. Pharmacol..

[39] Xiao X., Yang Y., Yang Y., Zhao G. (2026). Lactate Dehydrogenase B delactylation promotes gastric cancer metastasis via enhancing glutathione-mediated resistance to ferroptosis. Cell Biol. Toxicol..

[40] Li W., Liu T., Wu T., Cai T., Wang F., Zhang M. (2025). Posttranslational modifications in Helicobacter pylori-associated gastric pathogenesis: Bridging inflammation and carcinogenesis. Biochim. Biophys. Acta Rev. Cancer.

[41] Hanahan D., Weinberg R.A. (2011). Hallmarks of cancer: The next generation. Cell.

[42] Wang Z., Kang W., Li O., Qi F., Wang J., You Y., He P., Suo Z., Zheng Y., Liu H.M. (2021). Abrogation of USP7 is an alternative strategy to downregulate PD-L1 and sensitize gastric cancer cells to T cells killing. Acta Pharm. Sin. B.

[43] Zhou L., Wang H., Zhong M., Fang Z., Le Y., Nie F., Zhou J., Xiong J., Xiang X., Fang Z. (2022). The E3 Ubiquitin Ligase TRIM11 Facilitates Gastric Cancer Progression by Activating the Wnt/β-Catenin Pathway via Destabilizing Axin1 Protein. J. Oncol..

[44] Li L., Ng K.M., Shu X., Chai X., Wong K.Y., Srivastava G., Chan A.T., Chan W.Y., Tao Q. (2026). Loss of E3 Ubiquitin Ligase RINES via CpG Methylation Relieves Suppression of STAT3 and MYC, Facilitating Multiple Tumorigeneses. Adv. Sci..

[45] Bo S., You Y., Bai L., Liu S., Wang J., Jiang T., Tian S., Yan F., Cheng Y., Gao Y. (2026). FAM105A regulates the Hippo/YAP signaling pathway via deubiquitination to promote gastric cancer cell proliferation and migration. Exp. Cell Res..

[46] Wei G., Zhu X., Chen X., Lv J., Xiang F., Zhang M., Tao K., Wen W., He D., Wu X. (2026). Hypoxia-induced EPAS1/HIF2A-USP33-ATG101 axis drives oncogenic autophagy and peritoneal metastasis in gastric cancer. Autophagy.

[47] Ma C., Tian Z., Wang D., Gao W., Qian L., Zang Y., Xu X., Jia J., Liu Z. (2024). Ubiquitin-specific Protease 35 Promotes Gastric Cancer Metastasis by Increasing the Stability of Snail1. Int. J. Biol. Sci..

[48] Zhang T., Zheng J., Qiao L., Zhao W. (2022). Deubiquitinase USP13 promotes the epithelial-mesenchymal transition and metastasis in gastric cancer by maintaining Snail protein. Pathol. Res. Pract..

[49] Qian W., Li Q., Wu X., Li W., Li Q., Zhang J., Li M., Zhang D., Zhao H., Zou X. (2020). Deubiquitinase USP29 promotes gastric cancer cell migration by cooperating with phosphatase SCP1 to stabilize Snail protein. Oncogene.

[50] Zeng Z., Li Y., Zhou H., Li M., Ye J., Li D., Zhu Y., Zhang Y., Zhang X., Deng Y. (2024). System-wide identification of novel de-ubiquitination targets for USP10 in gastric cancer metastasis through multi-omics screening. BMC Cancer.

[51] Mao L., Yuan W., Cai K., Lai C., Huang C., Xu Y., Zhong S., Yang C., Wang R., Zeng P. (2021). EphA2-YES1-ANXA2 pathway promotes gastric cancer progression and metastasis. Oncogene.

[52] Tseng L.-L., Cheng H.-H., Yeh T.-S., Huang S.-C., Syu Y.-Y., Chuu C.-P., Yuh C.-H., Kung H.-J., Wang W.-C. (2020). Targeting the histone demethylase PHF8-mediated PKCα-Src-PTEN axis in HER2-negative gastric cancer. Proc. Natl. Acad. Sci. USA.

[53] Zang W., Ji Y., Zhou C., Li X., Lu Z., Zhang G., Chen Y., Xue W., Hu Y. (2026). Radiation-Induced Tumor-Intrinsic LTβR N-Glycosylation Suppresses Pyroptosis Through TRIM28-Mediated PCBP2 SUMOylation to Promote Gastric Cancer Radioresistance. Adv. Sci..

[54] Fang S., Jin G., Yan M., Song Y., Zhao S., Zhang C., Shao Y., Zhao K., Liu M., Wang Z. (2026). PHGDH phosphorylation mediated by WNK1 serves as a dual marker of metabolic vulnerability and responsiveness to oxaliplatin treatment. Proc. Natl. Acad. Sci. USA.

[55] Song J., Liu L., Wang F., Bao D. (2025). Targeting Ubiquitin-specific Protease 5 Overcomes Chemoresistance via Negatively Regulating p53 in Gastric Cancer. Curr. Mol. Med..

[56] Wu Z., Tu X., Zhu S., Jiang Q., Wang L., Tao K., Lou Z., Deng M., Zeng X. (2025). USP29 and SMURF1 orchestrate FSP1-mediated ferroptosis suppression to facilitate chemoresistance in gastric cancer. Nat. Commun..

[57] Zhang J., Tian T., Li X., Xu K., Lu Y., Li X., Zhao X., Cui Z., Wang Z., Zhou Y. (2025). p53 inhibits OTUD5 transcription to promote GPX4 degradation and induce ferroptosis in gastric cancer. Clin. Transl. Med..

[58] Zhang J., Han Y., Pan J., Liu Y., Cheng X., An J., Qiu J., Sun M., Yang J. (2025). High expression of SENP1 correlates with poor prognosis and immune evasion in gastric cancer. Int. Immunopharmacol..

[59] Tu C., Wu Z., Zhong X., Yang S., Chen X., Li S., Xu Y., Zuo Q. (2026). Endoplasmic reticulum stress-induced histone lactylation mediating immune escape of gastric cancer via PDIA6 overexpression in dendritic cells. Cell Death Dis..

[60] Zhou S., Xiao L., Hu L., Zuo F., Wang Y., Fei B., Dai J., Zhou X. (2025). CAFs promote immune evasion in gastric cancer through histone lactylation-mediated suppression of NCAPG ubiquitination. J. Transl. Med..

[61] Li B., Jiao L., Zhang Y. (2026). Pharmacological inhibition of histone Lactylation enhances immunotherapy efficacy in gastrointestinal cancers. Bioorg. Chem..

[62] Zeng K., Li W., Wang Y., Zhang Z., Zhang L., Zhang W., Xing Y., Zhou C. (2023). Inhibition of CDK1 Overcomes Oxaliplatin Resistance by Regulating ACSL4-mediated Ferroptosis in Colorectal Cancer. Adv. Sci..

[63] Li F., Dai Y., Tang C., Peng L., Huang H., Chen Y., Xu Y., Chen X., Wang Q., Lin Y. (2025). Elevated UBC9 expression and its oncogenic role in colorectal cancer progression and chemoresistance. Sci. Rep..

[64] Khan O.M., Carvalho J., Spencer-Dene B., Mitter R., Frith D., Snijders A.P., Wood S.A., Behrens A. (2018). The deubiquitinase USP9X regulates FBW7 stability and suppresses colorectal cancer. J. Clin. Investig..

[65] Jiang T., Liu J., Hu Q., Qi J., Liu B., Ma N., Xu Y., Zhang L., Chen J., Song J. (2026). Histone Lactylation Mediated by circRNA-Facilitated FXR1 Phase Separation Promotes Colorectal Cancer Progression. Cancer Res..

[66] Peng C., Wang T., Chen Z., Yang S., Jin C., Wang Y., Meng D., Peng W., Feng Y., Yang P. (2026). Histone Lactylation in Cancer-Associated Fibroblasts Induces Extracellular Matrix Remodeling and Immunosuppression to Promote Colorectal Cancer Progression. Cancer Res..

[67] Yi J.M., Kang T.H., Han Y.K., Park H.Y., Yang J.H., Bae J.H., Suh J.S., Kim T.J., Kim J.G., Cui Y.H. (2023). Human Neuralized is a novel tumour suppressor targeting Wnt/β-catenin signalling in colon cancer. EMBO Rep..

[68] Ye K., Wang P.C., Chen Y.X., Huang Q.Z., Chi P. (2024). E3 ubiquitin ligase BTBD3 inhibits tumorigenesis of colorectal cancer by regulating the TYRO3/Wnt/β-catenin signaling axis. Cancer Cell Int..

[69] Li W., Peng J., He J., Liao L., Kang D., Zhang W., Wang W., Wang R., Wang S., Liao Y. (2026). PKM2 Lactylation Promotes Colorectal Cancer Vasculogenic Mimicry and Bevacizumab Resistance by Facilitating FOSL1 Super-enhancer Formation. Cancer Res..

[70] Li W., Yuan W., Du Z., Wang X., Wang D., Xing S., Shen T., Lu C., Chen J., Yu A. (2026). AKR1B10 reprograms neutrophils by histone lactylation to foster immune evasion in KRASG12C mutation colorectal cancer liver metastasis. J. Exp. Clin. Cancer Res..

[71] Ye D.X., Wang S.S., Huang Y., Wang X.J., Chi P. (2021). USP43 directly regulates ZEB1 protein, mediating proliferation and metastasis of colorectal cancer. J. Cancer.

[72] Jiang M., Xiong C. (2025). USP7 accelerates colorectal cancer progression by up-regulating MYO6 through deubiquitination. Mutat. Res..

[73] Diefenbacher M.E., Popov N., Blake S.M., Schülein-Völk C., Nye E., Spencer-Dene B., Jaenicke L.A., Eilers M., Behrens A. (2014). The deubiquitinase USP28 controls intestinal homeostasis and promotes colorectal cancer. J. Clin. Investig..

[74] Choi B.J., Park S.A., Lee S.Y., Cha Y.N., Surh Y.J. (2017). Hypoxia induces epithelial-mesenchymal transition in colorectal cancer cells through ubiquitin-specific protease 47-mediated stabilization of Snail: A potential role of Sox9. Sci. Rep..

[75] Hong K.S., Ryu K.J., Kim H., Kim M., Park S.H., Kim T., Yang J.W., Hwangbo C., Kim K.D., Park Y.J. (2025). MSK1 promotes colorectal cancer metastasis by increasing Snail protein stability through USP5-mediated Snail deubiquitination. Exp. Mol. Med..

[76] Zhou Y., Wu J., Fu X., Du W., Zhou L., Meng X., Yu H., Lin J., Ye W., Liu J. (2014). OTUB1 promotes metastasis and serves as a marker of poor prognosis in colorectal cancer. Mol. Cancer.

[77] Sheng Z., Luo S., Huang L., Deng Y.-N., Zhang N., Luo Y., Zhao X., Chen Y., Li Q., Dai R. (2025). SENP1-mediated deSUMOylation of YBX1 promotes colorectal cancer development through the SENP1-YBX1-AKT signaling axis. Oncogene.

[78] Yu Y., Bao W., Chen J., Li Y., Gao Y. (2026). SENP3 drives colorectal cancer progression by enhancing GDF15 expression. Int. J. Color. Dis..

[79] Huang M., Qin Y., Yang Q., Chen X., Chen K. (2026). UHRF2 related to sumoylation: Assessment of its role in immune suppression and therapeutic potential in colorectal cancer. BMC Cancer.

[80] Cancer Genome Atlas Network (2012). Comprehensive molecular characterization of human colon and rectal cancer. Nature.

[81] Ma C.C., Jin C.Y., Guo N.J., Liu H.M., Yin Q.G., Wang Y.B., Liu Y.F., Zhang Y., Wang N., Lu G.L. (2026). A homologous cell membrane-coated peptide-based USP7 degrader enhances degradation efficiency and anti-tumor activity in gastric Cancer. Bioorg. Chem..

[82] Oliveira R.I., Guedes R.A., Salvador J.A.R. (2022). Highlights in USP7 inhibitors for cancer treatment. Front. Chem..

[83] Birappa G., Perumalsamy H., Hong S.-H., Gowda D.A.A., Chandrasekaran A.P., Karapurkar J.K., Rajkumar S., Balusamy S.R., Jayachandran A., Baek K.-H. (2026). Single-cell RNA sequence analysis reveals USP32 as a therapeutic target to mitigate PD-L1-driven colorectal tumorigenesis in vitro and in vivo. Theranostics.

[84] Dai Y., Luo X., Wu H., Luo Y., Deng Z., Wu J., Zhang L., Yan J. (2026). The role of deubiquitinase USP33 in colorectal cancer tumorigenesis and its potential as a therapeutic target predictor. Discov. Oncol..

[85] Gu L.H., Guo A., Ding Y.Y., Wang X.J., Zhang H.X., Duan W.L., Zhang B.G. (2025). Molecular mechanisms and potential targeting strategies of ubiquitin-proteasome system-mediated PD-1/PD-L1 ubiquitination in tumor immune suppression. Oncol. Rep..

[86] Tang G., Zuo H., Long R., Zhang C., Lv Y., Luo X., Qiu J., Zuo J. (2026). Lactylation enzymes in cancer: Mechanisms and novel therapeutic approaches (Review). Oncol. Lett..

[87] Fang C., Zhou S., Yu K., Zhang L., Lian W., Tie X., Chen S., Dai J., Li Q., Wang X. (2026). Lactate metabolism and lactylation in cancer: From pathogenesis to therapeutic advances. Signal Transduct. Target. Ther..

[88] Tan S., Zhang W., Ding Y., Liu J., Zhu M., Luo X., Cai J., Zeng H. (2026). The glycolysis-lactylation axis: A metabolic-epigenetic driver of immunosuppression and therapy resistance in cancer. Front. Immunol..

